# Needs-based human resources for health planning in Jamaica: using simulation modelling to inform policy options for pharmacists in the public sector

**DOI:** 10.1186/1478-4491-12-67

**Published:** 2014-12-06

**Authors:** Gail Tomblin Murphy, Adrian MacKenzie, Joan Guy-Walker, Claudette Walker

**Affiliations:** WHO/PAHO Collaborating Centre on Health Workforce Planning and Research, Dalhousie University, 5869 University Avenue, Halifax, NS B3H 4R2 Canada; Human Resource Management and Development, Jamaica Ministry of Health, 2-4 King Street, Kingston 10, Kingston, Jamaica

**Keywords:** Human resources for health, Health human resources, Health workforce planning, Jamaica, Pharmacists

## Abstract

**Background:**

Planning for human resources for health (HRH) is central to health systems strengthening around the world, including in the Caribbean and Jamaica. In an effort to align Jamaica’s health workforce with the changing health needs of its people, a partnership was established between Jamaican and Canadian partners. The purpose of the work described in this paper is to describe the development and application of a needs-based HRH simulation model for pharmacists in Jamaica’s largest health region.

**Methods:**

Guided by a Steering Committee of Jamaican stakeholders, a simulation modelling approach originally developed in Canada was adapted for the Jamaican context. The purpose of this approach is to promote understanding of how various factors affect the supply of and/or requirements for HRH in different scenarios, and to identify policy levers for influencing each of these under different future scenarios. This is done by integrating knowledge of different components of the health care system into a single tool that shows how changes to different parameters affect HRH supply or requirements. Data to populate the model were obtained from multiple administrative databases and key informants. Findings were validated with the Steering Committee.

**Results:**

The model estimated an initial shortage of 110 full-time equivalent (FTE) pharmacists in the South East Region that, without intervention, would increase to a shortage of about 150 FTEs over a 15-year period. In contrast to the relatively small impact of a large enrolment increase in Jamaica’s pharmacy training programme, interventions to increase recruitment of pharmacists to the public sector, or improve productivity - through, for example, the use of support staff and/or new technologies - may have much greater impact on reducing this shortage.

**Conclusions:**

The model represents an improvement on the HRH planning tools previously used in Jamaica in that it supports the estimation of HRH requirements based directly on measures of population health need. Both the profession (pharmacists) and country (Jamaica) considered here are under-studied. Further investments by Jamaica’s MoH in continuing to build capacity to use such models, in combination with their efforts to enhance health information systems, will support better informed HRH planning in Jamaica.

## Background

Jamaica is the largest English-speaking country in the Caribbean with a population of approximately 2.8 million. Recently reclassified as a lower middle-income country with a mean gross national income (GNI) of US$7,310 per capita, Jamaica spends roughly 5% of its gross domestic product (GDP) on health care [1]. Health care in Jamaica is financed through a mix of public and private sources, with approximately 56% of health care expenditures coming from public funds, 11% from private insurance, and 31% in the form of user fees and other out-of-pocket expenses [2]. The public health care system is administered through 4 Regional Health Authorities (RHAs) with a network of 24 hospitals and 348 primary health care centres across the island [1]. In addition to chronic shortages of funding and personnel - Jamaica’s combined density of physicians, nurses and midwives of 15 per 10,000 population is well below the regional average of 66 per 10,000 [3] - the public health care system is hindered by highly fragmented health information systems. The information available about the private sector is minimal and unreliable [1].

The shortages of human resources for health (HRH) in Jamaica mean that effective planning and management of scarce HRH is central to strengthening Jamaica’s health care system [4]. In order to address these challenges, policy-makers and decision-makers in Jamaica have recognized that the capacities of the health care workforce needs to be aligned with the needs of the population.

As noted in Jamaica’s Vision 2030 [4], 'one of the key advantages that a country can offer is the quality of its human capital. A well-trained workforce is emerging as one of the key drivers of a country's prosperity and competitiveness' (p. 64). Ensuring an adequate workforce within the health sector, in particular, has been identified as a national priority [5]. However, the types and numbers of HRH included in Jamaica’s current established posts, or cadres, are out of date, having not been updated since the 1970s [6]. As such, there is a strong desire among policy- and decision-makers in Jamaica for a means to estimate Jamaica’s health workforce requirements that is objective, based on current data, and considers the health care needs of the population as the basis for these requirements [5]. This challenging task is made easier when information on the health workforce and the population’s health needs is readily available to policy-makers. However, those responsible for the management of HRH in Jamaica are limited in their efforts by the availability and quality of planning tools.

Stemming from a partnership formed between the Jamaica Ministry of Health (MoH), Pan American Health Organization (PAHO), and the research team at the World Health Organization/Pan American Health Organization (WHO/PAHO) Collaborating Centre on Health Workforce Planning and Research at Dalhousie University, a needs-based approach to HRH planning originally developed by Canadian partners [7–9] was adapted to Jamaica’s unique context, beginning in late 2007. This partnership, supported by the Jamaica MoH, Health Canada and PAHO, was established in response to the Toronto Call to Action 2006 to 2015 [10] and the subsequently emphasized need to address the lack of alignment between the health workforce and population needs in Jamaica. The partnership is also aligned with the PAHO's 20 HRH Goals for the Next Decade [11] and the recently developed Road Map for HRH in the Caribbean [12]. The partnership has been guided by a Steering Committee of key stakeholders in HRH from across Jamaica. Members of this committee included representatives from the Jamaica MoH (including the executive office, HRH unit, planning unit, and epidemiology unit) and each of the RHAs, as well as the Ministry of Education, the Ministry of Finance, the Cabinet Office, the University of the West Indies (UWI), the University of Technology (UTech), Northern Caribbean University (NCU), the Statistical Institute of Jamaica (STATIN), and the Jamaica Employers’ Federation (JEF).

The primary objective of this partnership is to build capacity for needs-based HRH planning in Jamaica. One set of activities conducted in pursuit of this objective is the development of analytical and communication tools, in the form of simulation models, that can be used to support needs-based health workforce planning on an on-going basis. It is the intention of the partners to develop such tools for all Jamaica’s health professions. To date, simulation models for five professions in Jamaica - contact investigators, dentists, midwives, pharmacists, and psychiatrists - have been developed and delivered to the Jamaica Ministry of Health; these five were selected for an initial focus based on the perceived severity of shortages, policy and planning needs, and the availability of data.

Throughout the project, the Steering Committee provided validation of the model structure’s appropriateness to the Jamaican context, identified the professions to be modelled first, identified data sources, and insight into the interpretation of findings. It was also the Steering Committee which determined that the models should be designed at the RHA level because it is through the RHAs that the public health care system is administered. In addition, they determined that the models should be specific to HRH in the public sector, as these are the resources for which the RHAs are responsible - the RHAs’ influence over private sector practitioners is minimal. They further determined that, if resource and time constraints prohibited the development of models for all four RHAs, that the first models developed should apply to the South East RHA (SERHA), which is the largest in the country.

Pharmacists and pharmacy services are areas of particular interest for Jamaica’s MoH. Following the abolition of user fees for MoH services in 2008, patient loads across all public health facilities increased 30%. This increase in demand was more keenly felt at government-run pharmacies, however, where in addition to the removal of user fees, the list of drugs paid for where required by public funds was expanded by over 20% [13]; government expenditures on pharmaceuticals and medical supplies tripled between 2007 and 2008 [14]. No additional public sector pharmacists were on hand to meet this increase in demand. This shortage of personnel, in combination with shortages of some drugs, has contributed to wait times for having prescriptions filled that surpass 6 hours at some facilities [14].

The purpose of the work described in this paper is to describe the development and application of a needs-based HRH simulation model for pharmacists in the SERHA. The research questions guiding this work were the following:

-  How many public sector pharmacists will be available to provide services to residents of the SERHA in different future scenarios?
-  How many public sector pharmacists will be required to provide services to residents of the SERHA in different future scenarios?
-  What kinds of policy options may be most effective in minimizing any future ‘gap’ or difference between the numbers of public sector pharmacists available and required in the SERHA?

## Methods

The simulation modelling approach was informed by conceptual [7] and analytical [8] frameworks originally developed to inform HRH planning in Canada. It has been the work of the Jamaican Steering Committee and its partners at Dalhousie University to adapt this approach to suit the context of Jamaica. The adaptations required were largely related to the identification of appropriate data sources, as the Steering Committee viewed the general principles and structure of the models to be valid for Jamaica.

The purpose of this approach is not to predict the future, but rather to integrate knowledge of different components of the health care system into a single tool in order to a) promote understanding of how various factors affect the supply of and/or requirements for HRH, and b) identify policy levers for influencing each of these in Jamaica. The models are designed to enable Jamaica’s policy-makers to ‘rehearse’ potential policies by altering the value of model parameters and examining the estimated effects of such changes on the supply and requirements for a given type of HRH so as to evaluate the potential effectiveness of different HRH policies under different future scenarios.

**Figure 1:**
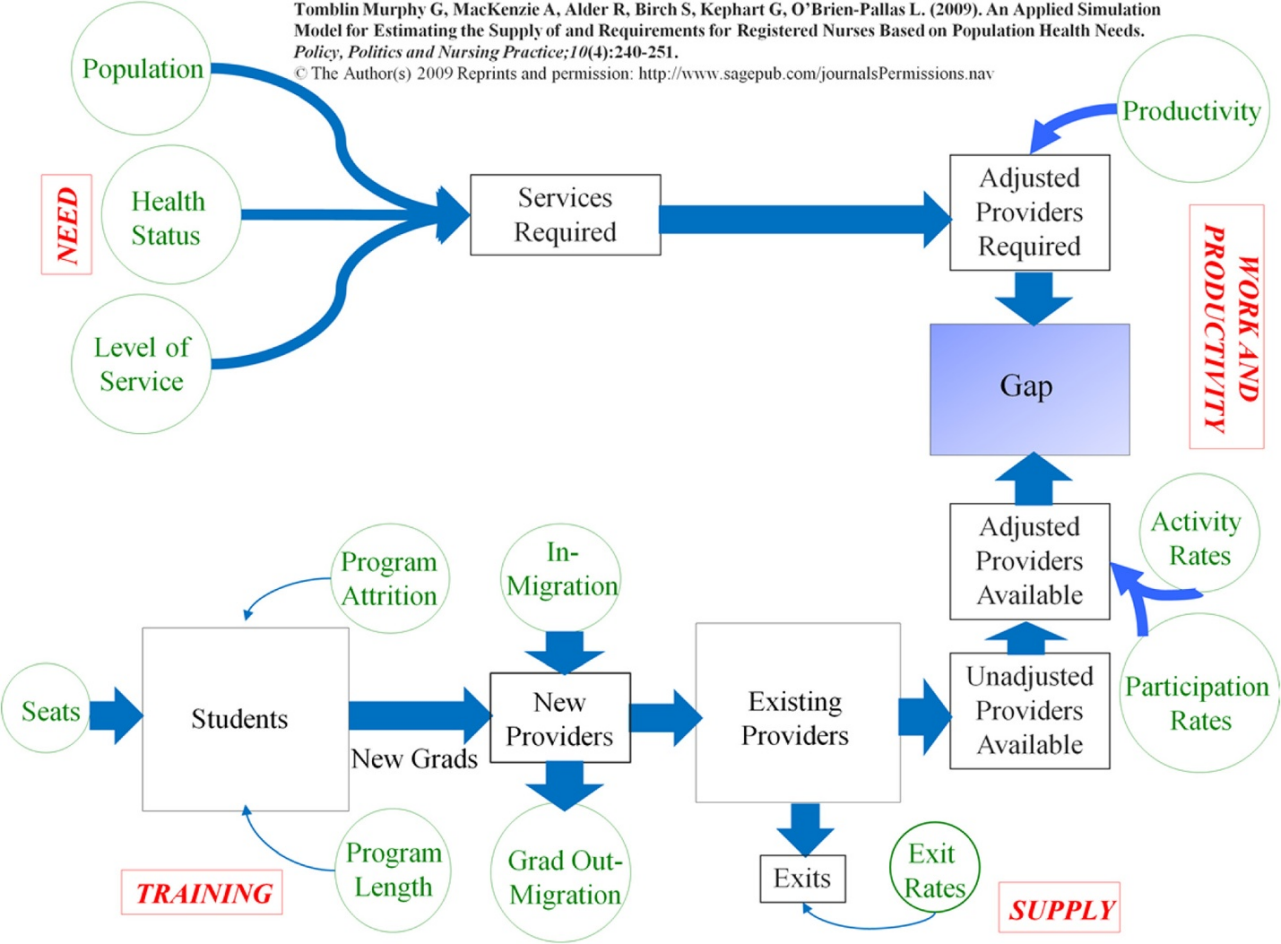
**Human Resources for Health (HRH) simulation model used in Jamaica.**

As noted above, for the purpose of this article, only the simulation modelling for pharmacists will be discussed, as the data pertaining to this profession lent themselves most readily to modelling. The general form of each of the models and the relationships between their components are depicted in Figure 1.

Each model consists of four modules: training, supply, work and productivity, and needs. Although these modules are identified separately for the sake of clarity, there are strong relationships between them. For example, the ‘outputs’ of the training module will affect those of the ‘supply’ module as providers complete their education and training and enter the workforce. Although applications of this general structure in Canada have been published previously, [15, 16] the functions of these modules are described individually below in terms of their application to pharmacists in Jamaica.

### The supply module

The purpose of the supply module is to estimate the future size of the supply of pharmacists in SERHA based on the *existing* supply, the number of *new providers* entering that supply, and the number of *exits* from that supply over time. The existing pharmacist supply is the quantity - or head count - of pharmacists living in SERHA who are potentially available to provide pharmacy services; this includes those who currently hold licenses to practise as pharmacists, regardless of whether or not they are currently doing so. The existing stock is specified by single year of age to allow for the ‘ageing’ of this population.

Pharmacists entering the existing stock are divided into two groups - *new graduates* of the pharmacy programme at UTech, and pharmacists beginning practice in SERHA who had previously been practising elsewhere, or not at all. The latter phenomenon, termed *in-migration*, would include pharmacists migrating from other jurisdictions, such as other Jamaican RHAs, or other countries.

Pharmacists exit the available supply for a variety of reasons such as retirement, relocation, or death. Since the number and type of these exits vary by age, changes in particular reasons for exits can be simulated by adjusting exit rates at different ages. For example, adjusting exit rates over age 50 or 55 can be used to simulate alternative retirement scenarios.

The combination of flows of pharmacists in and out of the existing supply determines whether the supply grows or declines. In the short term, the age distribution of this supply can exert a very powerful influence on its rate of growth or decline in size. For example, since rates of exit are greater at older ages, the total number of providers ‘lost’ will depend on the proportion of the supply in those age groups with the highest incidence of retirement.

### The training module

UTech’s pharmacy programme is the only one of its kind in the country and, therefore, represents an important source of additions to the supply of pharmacists in SERHA, and indeed to all of Jamaica. The training module focuses on estimating the flow of *new graduates* from such programmes - it can be adjusted to accommodate any additional pharmacist training programmes that may be developed - into the SERHA supply, which requires an understanding of the factors affecting that flow. The size of first-year enrolment, or number of *seats*, in the UTech programme is just one of several determinants of the flow of new pharmacy graduates. The flow of graduates will also depend on *programme length* (the distribution of years to graduation among cohorts of students), the *attrition rate* of the programme (the proportion of students who enrol in the programme but do not graduate) and the rate of entry to the regional supply by new graduates. The latter rate is determined by *graduate out-migration*; that is, the number of UTech pharmacy graduates who do not enter the SERHA workforce, but may instead, for example, begin practice in the private sector, or another region or country.

### The work and productivity module

The simulation model differentiates between the size of the pharmacist stock (the head count of licensed pharmacists) and the contributions of those pharmacists to the public health care system. The capacity of the pharmacist stock to meet requirements depends not only on the number of pharmacists, but also on the hours of pharmacy services they provide related to direct care of the public sector. For example, some are unemployed, others are employed but only work in the private sector, or are engaged exclusively in research, administration or education; these are deemed non-participants in direct patient care. Among those employed in the public sector (who are participants), the proportion of a full-time equivalent (FTE) employee each pharmacist represents (activity) can vary (for example, some work part-time hours, full-time or more than full-time hours). In this module, the number of licensed pharmacists living in the South East Region is converted from a head count to a measure of public sector FTEs by multiplying by the proportion who are employed in the public sector (*level of participation*) and, among those in the public sector, the proportion of an FTE the average pharmacist represents (*level of activity*). In addition, the number of dispensed items required to provide the desired level of service to the population according to the level and distribution of health within that population (from the needs module - described below) is converted to the number of pharmacists required to dispense those items by dividing by the average number of items a full-time pharmacist can reasonably be expected to dispense in a year (*productivity*). In short, this module translates the number of licensed pharmacists and the number of pharmacy services required into, respectively, the number of public sector FTE pharmacists required and available.

Changes in the distribution of participation and activity of pharmacists provide important mechanisms for meeting requirements for pharmacy services. Adding ‘sessions’ (over-time shifts), for example, increases the number of FTE pharmacists providing care, other things equal. However, this may also negatively affect the productivity of pharmacists, or the rates at which they exit the system. For example, excessive overtime hours have been linked to lower productivity rates and burnout, resulting in higher rates of exit from the stock of providers [17].

### The needs module

The needs module estimates the number of pharmacy services required to meet the health needs of SERHA’s population based on the first three components of the analytical framework [8]: population, health need, and level of service. More specifically, these estimates are based on three distinct factors: 1) the size and age/sex distribution of the SERHA population; 2) the distribution of chronic illness (including diabetes, cancer, chronic obstructive pulmonary disease, heart disease, hypertension, arthritis, mental health conditions including anxiety, depression, and schizophrenia, and renal disease) and infectious disease (including malaria, HIV/AIDS, influenza, and dengue fever) by age and sex within that population - which result in the need for pharmacy services; and 3) the number of pharmacy services to be provided to that population according to different levels of need - in this case, whether they have any of these conditions. Multiplying these three components yields the number of pharmacy services required to meet the health needs of SERHA’s population. Changes to any of these three factors - such as population growth and/or ageing, improvements or reductions in population health (and thus need for health care), or increasing or decreasing the planned levels of pharmacy service provision for different levels of need - will affect the estimated number of pharmacy services required for SERHA.

### Data sources and limitations

The sources of data used in the simulation of pharmacists are summarized in Table 1. Sources identified with plain text indicate that the data referred to came from administrative records. Those in *italicized* text indicate that administrative data was only partially available or that proxies were used where administrative data was not available - for example, estimates of the attrition rate from UTech’s pharmacy programme provided by the registrar and dean responsible for the programme.

**Table 1:** **Data sources and baseline values for Jamaica Human Resources for Health (HRH) simulation model for pharmacists**

Module	Data element	Data source	Baseline value
Training	Seats	UTech	85 new students per year
	Programme attrition	*UTech*	19% (81% graduation rate)
	Programme length	UTech	4 years
	Graduate out-migration	*UTech*	90%
Supply	In-migration	SERHA	0
	Existing provider stock	SERHA/UWI ERU	55
	Exit rates	*SERHA*	5% per year
Work and productivity	Participation rate	*SERHA*	55%
	Activity rate	SERHA	125% (average 50 hours/week)
	Productivity	*MoH*	16,800 items dispensed per FTE pharmacist per year
Needs	Population	STATIN	1.33 million
	Need	*MoH/STATIN*	Incidence/prevalence of major health conditions ranged from 0.01% (tuberculosis; rheumatic fever) to 0.9% (cancer)
	Level of service	*MoH/National Health Fund*	# prescriptions per recipient per year by condition ranged from 3 (arthritis) to 10 (psychosis)

*Abbreviations:* FTE, full-time equivalent; MoH, Ministry of Health; SERHA, South East Regional Health Authority; UTech, University of Technology; UWIERU, University of West Indies Epidemiology Research Unit.

Key challenges in obtaining certain data elements were mainly linked to two limitations of Jamaica’s current health information system (HIS): the lack of a unique identifier for health workers and the lack of a unique identifier for persons using the health system. The absence of the latter means that it is very difficult to determine what services individuals receive according to their health needs (levels of service). For example, if there were 100 hospital admissions among a population of 100 people over the course of a year, it is not possible to determine whether 1 person was admitted 100 times, 100 people were each admitted once, or any combination in between. The former limitation means it is difficult to establish what the current supply of health providers is, let alone to track flows of health care providers in and out of that supply. For example, if a large number of providers leave but are replaced by an equally large number of new ones, it would appear that the supply is static when in fact it has undergone a great deal of flux.

Estimating pharmacist entry and exit rates for SERHA, as well as activity levels, required the manual review of individual employment records, as these data are not kept in a longitudinal database. Estimating participation levels for SERHA pharmacists involved dividing the number of pharmacists employed by SERHA by the numbers of licensed pharmacists residing in SERHA (obtained from the Jamaica Database for Human Resources for Health developed by the Epidemiology Research Unit at the University of the West Indies).

The MoH has considerable administrative data on use of health care services, particularly those delivered within hospitals. However, these cannot provide accurate measures of the incidence and prevalence of various health conditions – which result in the need for health care services - in Jamaica, due to the lack of an individual identifier for patients and because many chronic conditions, such as arthritis, may not often require hospitalization. Estimates of population health need were, therefore, taken from data on incidence and prevalence of each of these conditions through population health surveys administered by STATIN and the Planning Institute of Jamaica (PIOJ). These surveys are designed to be representative of the entire country, and as such include persons who do not receive health care (thus providing a measure of unmet need) as well as those who do.

MoH administrative data is also not suitable for the estimation of levels of service - in the case of pharmacy services, the number of items dispensed per person according to their level of need. As above, this is because of the lack of a unique identifier for patients. For the purposes of this project, level of service data were instead provided by the National Health Fund (NHF), which administers a publicly-funded drug payment plan for which all Jamaicans with any of a range of diagnosed conditions are eligible [18]. Users of this programme can be uniquely identified in the NHF’s administrative data by their membership number, meaning it is possible to calculate the number of items dispensed for each user according to their particular health conditions, and thus estimate levels of service for each need group. It was the view of the Steering Committee that the number of items dispensed per person according to their level of need among NHF patients reflected an appropriate level of service for all Jamaicans because the drugs included under the NHF have been deemed to be the most appropriate to address the various conditions it covers, and because they can be filled at any pharmacy in Jamaica - public or private - and so are not as limited by the personnel shortages in the public sector.

Although the Jamaica MoH does not have complete data on the proportion of pharmacy services provided by the private sector, the model was initialized using the assumption that the public sector would be responsible for 30% of pharmacy services in SERHA. This value was estimated based on the ratio of publicly administered pharmacies to private ones of roughly 1:2. Although the model was developed to be specific to pharmacists working in the public sector, it can accommodate different assumptions about the proportion of required pharmacy services for which the public sector is to be responsible.

Challenges obtaining quality HRH data are prevalent for all types of HRH planning and research [19, 20]. Recognizing the need for a strengthened HIS in Jamaica, the MoH is working toward the development of a national strategic plan for HIS fortification and modernization [21]. The model is designed to be easily updated as improved or more current data become available -for example, as newer iterations of population health surveys are completed - or as various planning assumptions change over time. For example, if it is determined that the level of pharmacy services to be provided to Jamaicans should be increased or otherwise changed, the model can be adjusted to reflect this and determine the implications of such a change for pharmacist requirements. Similarly, if it is determined that the proportion of pharmacy services to be provided by the public sector should change, the model’s input data can be easily updated to reflect this.

UTech’s pharmacy programme is 4 years in length with a typical annual enrolment of 85 new students. Of those 85 students who enter each year, an average of 70 complete the programme successfully (an attrition rate of roughly 18%). Of those who graduate from the programme, between 5% and 10% enter the public service as pharmacists, with the remainder entering the private sector or leaving to practise overseas.

SERHA records indicate there as of 2008 there was almost no in-migration of pharmacists to practise for the RHA, either from other RHAs or other countries. According to the Jamaican HRH database established by the Epidemiology Research Unit at the University of the West Indies -Mona campus, there were 55 pharmacists practising within the South East RHA (including those working in the public and private sectors), with an average age of 36. SERHA records indicated that 30 of those pharmacists were employed in the public sector (for a participation rate of 55%), and that between regular hours and overtime ‘sessions’, those 30 pharmacists averaged roughly 50 hours of work per week (for an activity rate of 125%). SERHA records also indicated that those pharmacists filled close to 629,000 prescriptions that year, for an average productivity of roughly 16,800 prescriptions filled per full-time pharmacist per year.

SERHA has a population of 1.33 million. Data from population health surveys conducted by STATIN showed that crude incidence and prevalence rates for a variety of chronic and infectious conditions ranged from a low of 0.1% for tuberculosis and rheumatic fever up to 0.9% for cancer. Data from the NHF on the number of prescriptions received per person by individuals with those conditions show that these values ranged from an average of three per year for people with arthritis to ten per year for people with psychoses.

These data were used to populate the model and create a ‘baseline’ scenario showing how the estimated shortage of pharmacists in SERHA would change over a 15-year period if current rates of inflows and outflows were maintained. This initial scenario was shared with the Steering Committee to promote discussion of various policy scenarios that could be used to offset such a shortage. These scenarios were then simulated using the model to compare their relative effectiveness in reducing future pharmacist shortages in the South East Region.

## Results

In 2008, there were 37.5 FTE pharmacists working in the public sector in SERHA. To provide the pharmacy services required by the SERHA population (based on the estimated prevalence of chronic conditions and incidence of infectious diseases from the latest population health survey) in 2008, approximately 150 FTE pharmacists would be required. Hence the model estimated an initial shortage of 110 FTE pharmacists in SERHA in 2008.

**Figure 2:**
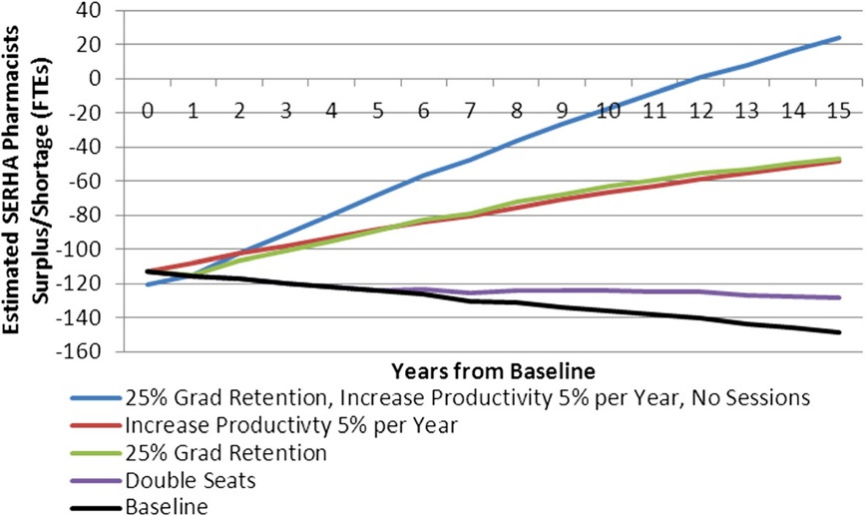
**Simulated South East Regional Health Authority (SERHA) pharmacist gap under various policy scenarios.**

Model results further indicate that, without any policy intervention, the estimated shortage of SERHA pharmacists would continue to increase steadily over the next 15 years (Figure 2 - black curve), to a shortage of about 150 FTEs by 2023. The gap increases in this scenario because, even if the distribution of health within the SERHA population stays the same, more pharmacists would be required to deliver the desired level of service to a growing population. Due to very low levels of recruitment of new pharmacists to the public sector, SERHA’s supply of public sector pharmacists will not increase fast enough to keep pace with these growing service requirements.

The policy scenarios suggested by the Steering Committee to address this estimated shortage included an increase in enrolment in UTech’s pharmacist programme, an increase in the proportion of UTech graduates entering the public sector, and increased productivity of practising pharmacists (Figure 2).

In contrast to the relatively small impact of a large (100%) seat increase (denoted by the purple curve in Figure 2), simulation results suggest that increasing the portion of pharmacy graduates who enter the public sector workforce to 25% (green curve) would dramatically reduce the shortage of pharmacists in SERHA. Similarly, results suggest that increases in the productivity of pharmacists could potentially reduce the SERHA pharmacist shortage dramatically; a 5% annual increase (red curve) would cut the shortage by approximately two thirds over 15 years. Further, results suggest that if both of the above policy scenarios could be achieved together - that is, if 25% of pharmacy graduates could be retained by the public sector *and* the productivity of public sector pharmacists could be increased by 5% each year, then the shortage of pharmacists in SERHA would be reduced to the point that, within 15 years, the supply of pharmacists in the region would be sufficient to meet the needs of persons who rely on public sector pharmacies without the use of ‘sessions’, or overtime work by pharmacists (Figure 2 - blue curve).

## Discussion

When faced with an HRH shortage, the first instinct of policy-makers is often to identify an increase in education and training seats as the solution. However, there are several reasons why seat increases tend not to solve HRH shortages. First, there is a considerable delay between the implementation of a seat increase and the first ‘extra’ providers that result from it; in the case of Jamaica’s pharmacists, even doubling the number of seats at UTech’s pharmacy programme produces no additional pharmacists for at least 4 years - the length of the programme. Second, the number of additional graduates is initially small relative to the number of existing providers; it takes several years for these ‘extra’ providers to grow to add significantly to the existing supply. Third, not all of these additional seats will translate into additional providers for the jurisdiction, as some students will not complete the programme, some graduates will leave the RHA or the country, and some who stay will not work in the public sector. In the case of Jamaica pharmacists, only about 5 to 10% of the graduates of UTech’s pharmacy programme enter the public sector workforce. Thus it is not surprising that even the dramatic 100% pharmacy seat increase simulated shows little impact on the shortage of pharmacists in SERHA (Figure 2).

In contrast to the relatively small impact of a large seat increase, simulation results suggest that increasing the portion of pharmacy graduates who enter the public sector workforce to just 25% would dramatically reduce the shortage of pharmacists in SERHA (Figure 2). Although this would reduce the flow of new graduates to the private sector from current levels, there would still be considerably fewer new graduates entering the public sector than the private sector in this scenario. General HRH recruitment and retention literature [22–25], as well as reports specific to the planning for pharmacy services [26–28], suggest that such an improvement could be achieved through initiatives designed to enhance the appeal of the public sector [29], such as better working conditions, improved financial incentives, opportunities for professional development, or making some amount of public sector service a condition of registration for pharmacists. Financial incentives that have been successful for nurses in Jamaica include providing health insurance, paid vacation and transportation allowances [30]. Similarly, results suggest that increases in the productivity of pharmacists could potentially reduce the SERHA pharmacist shortage dramatically; a 5% annual increase would cut the shortage by approximately two thirds over 15 years. Productivity improvements in pharmacy services have been shown to be achievable through means such as investments in support staff (for example, pharmacist assistants) [27] or enhanced equipment or technology such as computerized order entries in hospital settings [31] and pre-packaging of medications [28, 29, 32]. The combined potential of these improvements to eliminate the need for overtime ‘sessions’ by pharmacists is particularly important given concerns regarding high pharmacist workloads and associated dispensing errors [33–35].

Additional policies that could be considered include, for example:

-  Exploring options for training experienced pharmacy technicians as pharmacists (which would increase the pharmacist supply)
-  Changing the number of items to be dispensed to address different health conditions in hopes of achieving better health outcomes (changing the level of service)
-  Establishing some type of bonding programme where some or all of some pharmacy students’ training costs are borne by the MoH in exchange for some agreed upon term of service in the public sector after the students graduate (which would increase retention of graduates)
-  Enhanced surveillance and management of infectious diseases such as dengue fever and malaria to reduce their incidence (and, by extension, the associated need for pharmacy services)

After considering the findings from this study presented at a meeting of its senior directors, Jamaica’s MoH reconsidered plans to increase enrolment in the UTech pharmacy programme as a means of addressing the pharmacist shortage in favour of investing in strategies to increase the attractiveness of public sector health care positions, such as increased salaries. It will be important for the MoH to work with its RHA and pharmacy partners to continue to monitor the status of Jamaica’s pharmacist workforce over time in order to assess the impacts of these strategies. As part of its on-going efforts to improve its HRH planning, the MoH has ensured that over two dozen members of its own personnel and RHA staff have been trained in the use of the simulation models. This investment will help to facilitate the on-going monitoring and evaluation of its planning for pharmacists and other professions.

Although the model is specified at the RHA level, the scenarios considered to address SERHA’s pharmacy shortage have national implications. Because Jamaica has only one pharmacist training programme, increasing its output affects not only the pool of potential new personnel available to the South East Region but the other three as well. Similarly, some means of improving recruitment of graduates into the public sector - such as increasing wages - would have to be implemented nationally as opposed to by individual regions. Further, increases in public sector wages would likely be an important strategy to consider for other critical health professions as well.

The model is designed to focus on deterministic relationships between variables while being adaptable to different professions and contexts. As such, it is not designed to encapsulate the entire practice environment of the professions being modelled, since the specific mathematical relationship between, for example, the physical layout of pharmacies and the productivity of pharmacists is not precisely known. The model can, however, be used to simulate the impacts of changes to that environment - such as changes to pharmacy layouts, or new technologies - by adjusting the anticipated productivity based on the change being considered.

In addition to the data limitations discussed above, it must also be acknowledged that several years have passed since they were collected in 2011. At that time, the most recent year for which a full range of acceptable data could be obtained was 2008. It is nonetheless the view of the authors that, given the comparative lack of representation of either Jamaica (relative to other countries in the Americas) or pharmacists (relative to doctors and nurses, for example) in the HRH literature, this approach and its findings, although somewhat dated, represent an important contribution to the existing body of evidence.

## Conclusion

A set of simulation models has been developed to inform HRH planning and policies in Jamaica. Although there is room to improve the data used to populate them, the models represent a method of estimating HRH requirements that explicitly incorporates data on measures of population health as well as planned levels of service provision according to different levels of health. In this way it is considerably more robust than the existing system, which is based on outdated cadres. The simulation model structure and input data are described in detail, and the results provide important insights into the determinants and dynamics of the supply of and requirements for an understudied profession (pharmacists) in an understudied country (Jamaica).

The shortage of pharmacists in Jamaica is not a problem of insufficient training capacity, but more of insufficient numbers of those pharmacists being produced entering the public sector. This situation emphasizes the importance of an attractive work environment for ensuring an adequately staffed public health care system. In this respect, the findings are consistent with key recommended actions in the workforce reports released by the International Pharmaceutical Federation [28, 29] that call for improved partnerships across organizations and sectors, enhanced HRH information systems and evidence-based strategic workforce plans to ensure an adequate pharmacy workforce as a key component of a broader adequate health care system.

Further investments by Jamaica’s MoH in building national capacity to use such models, in combination with their efforts to enhance the country’s HIS, will support on-going improvement of the health workforce models and their input data, which will in turn allow for better informed HRH planning in Jamaica. More broadly, as public sector pharmacy services in Jamaica are better understood - for example, by studying the factors that influence pharmacists’ productivity - further enhancements to the model can be considered to take advantage of improved evidence by explicitly incorporating such factors into its structure.

Future work in this area, in addition to updating the models with more recent data, will focus on discussing with Jamaican stakeholders an expanded range of policy scenarios, reflecting the country’s current context and challenges, for simulation. These could include, for example, the impacts of future changes to the health of the SERHA population, or to the portion of pharmacy services to be provided by the public sector. Additional policies that could be considered include amending existing legal and regulatory frameworks to recognize and define the roles and standards of practice of pharmacy technicians and exploring further private-public partnerships for the provision of pharmacy services, such as through the NHF.

## Abbreviations

FTE: full-time equivalent
GDP: gross domestic product
GNI: gross national income
HIS: Health Information System
HRH: Human Resources for Health
JEF: Jamaica Employers’ Federation
MoH: Ministry of Health
NCU: North Caribbean University
PAHO: Pan American Health Organization
UWI: University of the West Indies
PIOJ: Planning Institute of Jamaica
RHAs: Regional Health Authorities
SERHA: South East Regional Health Authority
STATIN: Statistical Institute of Jamaica
UTech: University of Technology, Jamaica
UWI: University of the West Indies UWIERU, University of West Indies Epidemiology Research Unit
WHO: World Health Organization.
